# Supplementary material for: Immunophenotypic and Functional Interindividual Variability in Banked Cord Blood Cells: Insights for Advanced Therapies
Source: Int J Mol Sci. 2025 Jan 30;26(3):1208. doi: 10.3390/ijms26031208 (PMC11818680; doi:10.3390/ijms26031208)
Supplement: Supplementary file 1 [file ijms-26-01208-s001.zip › ijms-3394654-supplementary.pdf]

Supplementary Tables

Table S1. Description of the Antibody panel used: Acute Leukemia Orientation Tube (ALOT), to identify and quantify the immature hematopoietic cell population.

**Fluorochromes**	**V450**	**V500**	**FITC**	**PE**	**PerCPCy5.5**	**PECy7**	**APC**	**APCH7**
**Markers**	CyCD3	CD45	CyMPO	CyCD79a	CD34	CD19	CD7	SmCD3
**Volume**	7 µl	5 µl	20 µl	20 µl	7 µl	5 µl	5 µl	3 µl

Abbreviations: FITC, fluorescein isothiocyanate; PE, phycoerythrin; PerCP, peridinin-chlorophyll-protein; Cy5.5, cyanin5.5; Cy7, cyanin7; APC, allophycocyanin; Cy, cytoplasmic; Sm, surface membrane.

Table S2. General Description of the Antibody Panel used to identify and quantify to T- Acute Lymphoblastic Leukemia (T-ALL)

**Fluorochromes**	**V450**	**V500**	**FITC**	**PE**	**PerCPCy5.5**	**PECy7**	**APC**	**APCH7**
**Markers**	cyCD3	CD45	CD2	CD117	CD4	CD8	CD7	SmCD3
**Volume**	7 µl	5 µl	5 µl	5 µl	4 µl	5 µl	5 µl	3 µl

Table S3. General Description of the Antibody Panel used to identify and quantify B-cell precursors for Acute Lymphoblastic Leukemia (ALL) – (BCP-ALL)

**Fluorochromes**	**V450**	**V500**	**FITC**	**PE**	**PerCPCy5.5**	**PECy7**	**APC**	**APCH7**
**Markers**	CD20	CD45	CD58	CD66	CD34	CD19	CD10	CD38
**Volume**	5 µl	5 µl	7 µl	20 µl	7 µl	5 µl	5 µl	3 µl

Table S4. General Description of the panel used to identify and quantify the Neutrophil maturation.

**Fluorochromes**	**V450**	**V500**	**FITC**	**PE**	**PerCPCy5.5**	**APC**	**APCH7**
Markers	HLA-DR	CD45	CD61	CD34	CD117	CD11b	CD10
Volume	5 µL	5 µL	7 µL	7 µL	5 µL	5 µL	5 µL

Table S5. General Description of the Antibody panel used to identify and quantify the Monocytic maduration.

Fluorochromes	V450	V500	FITC	PE	PerCPCy5.5	APC	APCH7
Markers	HLA-DR	CD45	CD35	CD46	CD117	IREM 2	CD14
Volume	5 μL	5 μL	5 μL	10 μL	5 μL	5 μL	5 μL

Table S6. UCB cellular $CD45^+$, $CD34^+$ viability, concentration, $CD45^+$ and $CD34^+$ viable events using ISHAGE protocol and eClone [13] after thawing (AT). Same samples from experiments shown in Table 1.

Donor	$CD45^+$ cells viability (%) AT	$CD34^+$ cells viability (%) AT	[$CD45^+$ viable] (cells/ μL) AT	[$CD34^+$ viable] (cells/ μL) AT	$CD45^+$ viable events AT	$CD34^+$ viable events AT	eClone (%) AT
1	31.6	35.37	1017.04	4.81	30679	145	33.3
2	54.5	71.23	2338.14	8.26	70774	250	44.3
3	58.6	77.2	2463.6	10.27	71501	298	39.3
4	49.2	60.87	1471.89	2.84	43493	84	37.7
5	46.6	66.53	2121.6	5.72	60450	163	28.3
6	36.6	63	794.47	6.05	27042	206	33.3
7	38.1	63.2	1429.49	4.76	47418	158	39.1
8	54.2	48.46	1350.26	9.51	35786	252	24.8
9	54.2	50.96	2160.73	13.99	69511	450	27.6
10	34.2	42.67	527.54	2.11	16010	64	7.7
11	43	56.61	1667.55	11.88	52902	377	30.2

Table S7. UCB cellular $CD45^+$, $CD34^+$ viability using ISHAGE protocol [13] after thawing (AT). Same samples from migration experiments, shown in Figures 11 and 12, were tested.

Donor	Total $CD45^+$ cells ($x10^7$) AT	Total $CD34^+$ cells ($x10^5$) AT	$CD45^+$ cells viability (%) AT	$CD34^+$ cells viability (%) AT
4	6.3	5.8	88	93
8	5.4	2.6	91	97
11	4.5	5.7	90	95
43	3.5	3.1	78.3	89.6
44	4.2	4.4	86.7	94.2
46	6.6	5.0	92.8	88.9

Supplementary Figures

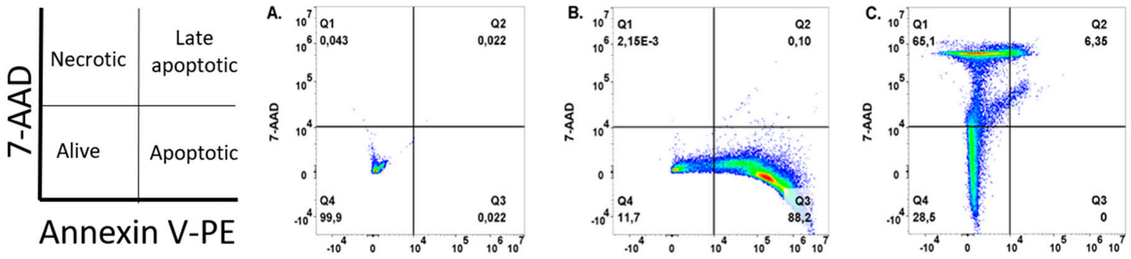

Figure S1. Flow cytometry analysis of Annexin V-PE and 7-AAD that shows viable, apoptotic and necrotic cells, as described in the materials and methods section. (**A**) Viable cells. (**B**) Apoptotic cells. (**C**) Necrotic cells.

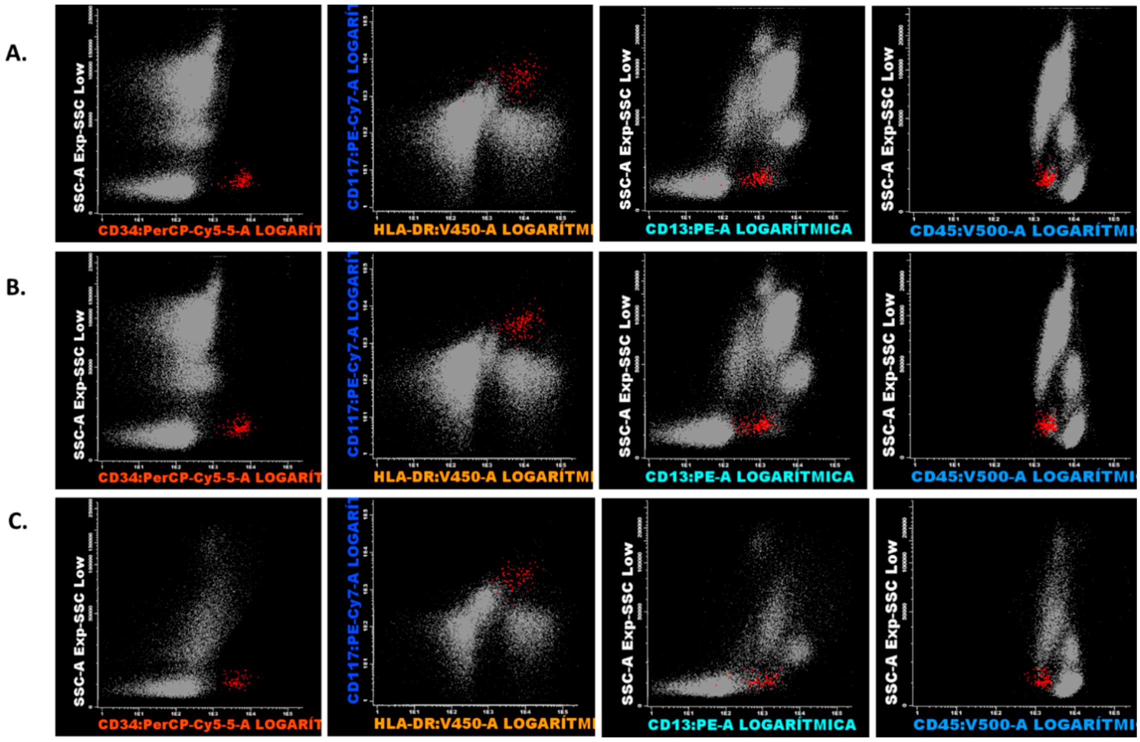

Figure S2. Flow cytometry analysis of CD117, CD13, CD45, and HLA-DR marker expression in myeloid CD34+ cells in umbilical cord blood samples under three experimental conditions. Immunophenotypic analysis was conducted using Infinicyt™ (Cytognos SL) software on one representative sample from the three experimental conditions: fresh sample (panel (**A**)), after VR (panel (**B**)), and after thawing (panel (**C**)). CD34+ cells (shown in red) displayed expression of CD117, CD13, CD45 (dim), and HLA-DR. In panel (**C**), it is observed that the sample after thawing shows low Side-Scatter in the granulocytic lineage.
